# TLR2 Signaling is Required for the Innate, but Not Adaptive Response to LVS *clpB*

**DOI:** 10.3389/fimmu.2014.00426

**Published:** 2014-09-05

**Authors:** Lydia M. Roberts, Hannah E. Ledvina, Gregory D. Sempowski, Jeffrey A. Frelinger

**Affiliations:** ^1^Department of Immunobiology, University of Arizona, Tucson, AZ, USA; ^2^Duke Human Vaccine Institute, Duke University, Durham, NC, USA

**Keywords:** *Francisella tularensis*, TLR2, clpB, T-cells, innate immunity, lung, intranasal

## Abstract

Toll-like receptor 2 (TLR2) is the best-characterized pattern-recognition receptor for the highly pathogenic intracellular bacterium, *Francisella tularensis*. We previously identified a mutant in the live vaccine strain (LVS) of *Francisella*, LVS *clpB*, which is attenuated, but induces a protective immune response. We sought to determine whether TLR2 signaling was required during the immune response to LVS *clpB*. TLR2 knock-out (TLR2 KO) mice previously infected with LVS *clpB* are completely protected during a lethal challenge with LVS. Furthermore, the kinetics and magnitude of the primary T-cell response in B6 and TLR2 KO mice are similar indicating that TLR2 signaling is dispensable for the adaptive immune response to LVS *clpB*. TLR2 signaling was important, however, for the innate immune response to LVS *clpB*. We identified three classes of cytokines/chemokines that differ in their dependence on TLR2 signaling for production on day 3 post-inoculation in the bronchoalveolar lavage fluid. IL-1α, IL-1β, IL-2, IL-17, MIP-1α, and TNF-α production depended on TLR2 signaling, while GM-CSF, IFN-γ, and VEGF production were completely independent of TLR2 signaling. IL-6, IL-12, IP-10, KC, and MIG production were partially dependent on TLR2 signaling. Together our data indicate that the innate immune response to LVS *clpB* requires TLR2 signaling for the maximal innate response, whereas TLR2 is not required for the adaptive immune response.

## Introduction

Germline-encoded pattern recognition receptors (PRRs) recognize conserved microbial components and initiate innate immune responses [reviewed in Ref. (1)]. Toll-like receptors (TLRs) are one class of PRR. TLR2 is the best-characterized PRR for the highly pathogenic, intracellular bacterium *Francisella tularensis*. TLR2 recognizes triacyl and diacyl lipoproteins when in complex with TLR1 or TLR6, respectively. Three TLR2 ligands have been identified in *Francisella*: LpnA (also known as Tul4), FTT_1103, and FTL_0645 (2–4). Ligand engagement of TLR2 leads to an association between TLR2’s Toll/IL-1R intracellular domain and MyD88 (5). MyD88 then recruits and activates IL-1 receptor-associated kinase 4 and TNFR-associated factor 6, which leads to downstream NF-κB activation and finally pro-inflammatory cytokine production (5). TLR2 knock-out (TLR2 KO) mice are more susceptible to wild-type *Francisella* with increased bacterial burdens and decreased mean time to death (6, 7). The increased susceptibility of TLR2 KO mice is likely due to the requirement for TLR2 signaling during the innate immune response to *Francisella* (6–13). For example, TLR2 KO peritoneal macrophages or bone marrow-derived dendritic cells (DCs) fail to make pro-inflammatory cytokines such as TNF-α, IL-12, and IL-6 (7, 8, 10).

One hallmark of pneumonic tularemia caused by wild-type *Francisella* is the near absence of an innate immune response in the lung despite high bacterial burdens (14). An in-frame deletion of the *clpB* gene in the live vaccine strain (LVS) of *F. tularensis* subsp. *holartica* results in bacteria that lack the ability to inhibit host innate immune (15). ClpB is a highly conserved chaperone protein of the AAA+ superfamily of ATPases, which mediate protein disaggregation (16). ClpB has not been shown to be a TLR2 ligand, nor has it been shown to affect the expression of identified TLR2 ligands (17). Intranasal inoculation of C57Bl/6J and BALB/cJ mice with LVS *clpB* significantly increases the concentration of pro-inflammatory cytokines and chemokines in the bronchoalveolar lavage fluid (BALF) 3 days post-inoculation compared to LVS inoculated mice (15). Despite a robust innate immune response during LVS *clpB* infection, adaptive immunity is required for bacterial clearance and the frequency of IFN-γ producing CD4^+^ and CD8^+^ T-cells is similar in mice inoculated with LVS or LVS *clpB* (15). We and others have demonstrated that vaccination with *clpB* mutants in both LVS and the highly virulent *F. tularensis* subspecies *tularensis* (SchuS4) provide protection during lethal, wild-type challenge (15, 17–19).

Due to the well-characterized role of TLR2 during the immune response to wild-type *Francisella*, we sought to determine whether TLR2 was required during the immune response to LVS *clpB*. TLR2 KO mice were able to clear LVS *clpB*; clearance, however, was delayed compared to B6 mice inoculated with LVS *clpB*. Additionally, TLR2 KO mice previously infected with LVS *clpB* survived lethal LVS challenge. The ability of TLR2 KO mice to survive a lethal secondary challenge was not surprising given that the T-cell response in B6 and TLR2 KO mice was similar on days 7 and 10 post-inoculation during the primary infection. Together, these data indicated that TLR2 signaling is dispensable during the primary and secondary T-cell response. However, TLR2 signaling was required for the maximal innate immune response to LVS *clpB*. We identified three classes of cytokines and chemokines in the BALF that differed in their requirement of TLR2 signaling for production on day 3 post-inoculation (TLR2 independent, TLR2 dependent, and TLR2 partially dependent). Together, these data indicated that while TLR2 is critical during the innate immune response, TLR2 signaling is dispensable during the primary adaptive immune response and a secondary challenge.

## Materials and Methods

### Bacteria

*Francisella tularensis* subspecies *holarctica* LVS with an in-frame deletion of *clpB* (FTL_0094) was generated as previously described (15). Wild-type LVS was obtained from the CDC (Atlanta, GA, USA). Bacteria were grown at 37°C on chocolate agar supplemented with 1% IsoVitalex (Becton-Dickinson). Bacterial inoculations were prepared by re-suspending bacteria from a lawn grown on chocolate agar in sterile PBS at an OD_600_ = 1 (equivalent to 1 × 10^10^ CFU/mL). The number of viable bacteria was determined by serial dilution and plating on chocolate agar.

### Mice

C57Bl/6J (B6) and B6.SJL-*Ptprc^a^Pepc^b^*/BoyJ (B6-CD45.1), and B6.129-*Tlr2^tm1Kir^*/J (TLR2 KO) mice were obtained from The Jackson Laboratory (Bar Harbor, ME, USA). TLR2 KO mice were bred in-house and were age-matched with vendor-purchased B6 mice. Female B6 and TLR2 KO mice were between 6 and 10 weeks old at the time of inoculation. All mice were housed in specific pathogen-free conditions at the University of Arizona in accordance with the Institutional Animal Care and Use Committee (IACUC).

### Inoculation of mice

Mice were anesthetized with 575 mg/kg tribromomethanol (Sigma) and intranasally inoculated with 5 × 10^4^ CFU LVS *clpB*. For lethal LVS challenge experiments, mice were anesthetized with 0.25 mL of 7.5 mg/mL ketamine and 0.5 mg/mL xylazine cocktail in PBS and intranasally inoculated with 5 × 10^3^ CFU (5 × LD_50_) LVS 35 days after the initial sub-lethal infection. Mice were weighed daily and sacrificed if they lost more than 25% of their starting weight.

### Bacterial burden determination

Spleen, liver, and lung tissue were homogenized in sterile PBS using a Biojector (Bioject). Ten-fold serial dilutions of tissue homogenates were made using PBS and plated on chocolate agar. Resulting colonies were counted 72 h later. The limit of detection is 50 colony forming units (CFU) per organ.

### Spleen, lung, and bronchoalveolar lavage cell isolation

Spleens and lungs were harvested from mice and processed into single-cell suspensions as previously described (15). BALF was collected as previously described (15). Cells were removed from the BALF using centrifugation and resulting supernatant was stored at −80°C for multiplex cytokine/chemokine profiling.

### Antibodies

The following directly conjugated antibodies were used for analyzing cells in the BALF: CD3 Pacific Blue (17A2; Biolegend), CD11b V500 (M1/70; BD), CD11c PE-Cy7 (N418; Biolegend), CD19 PerCP-Cy5.5 (6D5; Biolegend), F4/80 PE (BM8; Biolegend), and GR-1 AF700 (RB6-8C5; Biolegend). BALF cells were stained with 10 μg/mL AF350 succimidyl ester (Life Technologies) to distinguish live and dead cells prior to staining with surface antibodies. Antibodies used for intracellular cytokine staining (ICS) were the same as previously described (15).

### Cytokine/chemokine quantification

A multiplex luminex bead-based approach was used to quantify cytokines/chemokines in the BALF as described (15). A 20-analyte assay panel was performed according to the manufacturer’s protocol (Life Technologies) using a BioPlex array reader (Bio-Rad Laboratories) in the Duke Regional Biocontainment Laboratory Immunology Unit (Durhan, NC, USA).

### Intracellular cytokine staining

Intracellular cytokine staining was performed as previously described (15). Briefly, B6-CD45.1 splenocytes were inoculated with LVS at an MOI of 200:1. Two hours post-inoculation, cells were washed and fresh medium containing 5 μg/mL gentamicin (Sigma) was added. Infected splenocytes were incubated overnight in the presence of gentamicin. Infected splenocytes were washed extensively and then cultured at a 1:1 ratio with cells isolated from the spleen and lung of infected mice for 24 h. A total of 10 μg/mL Brefeldin A (Sigma) was added during the last 4 h of culture to stop cytokine secretion. Flow cytometry data were analyzed as previously described using FlowJo v10.0.6 (Treestar) (15).

### Statistical analysis

A one-way ANOVA with Tukey’s post-test was used for BALF cytokine and chemokine concentrations. A Mann–Whitney test was used for ICS data to compare B6 and TLR2 KO mice on days 0, 7, or 10 post-inoculation. Bacterial burdens were log transformed and then a Student’s *t*-test was applied. GraphPad Prism (v5.04) was used for analysis. Significance levels are indicated in the figures as follows: *P < 0.05, **P < 0.01, ***P < 0.001, and ****P < 0.0001.

## Results

### TLR2 KO and B6 mice have different disease courses when inoculated with LVS *clpB*

**Figure 1:**
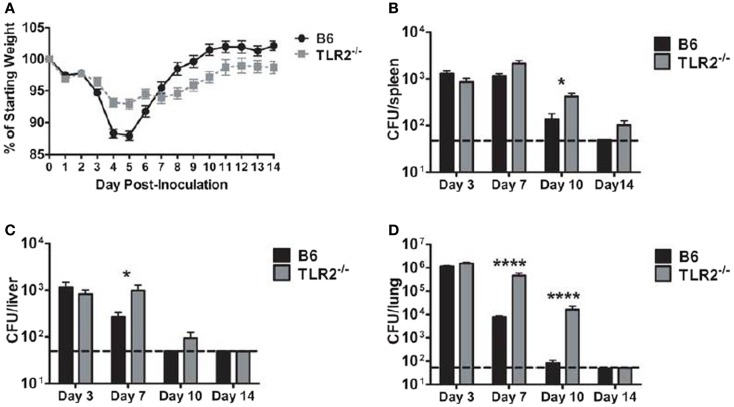
**LVS *clpB* clearance is delayed in TLR2 KO mice**. B6 or TLR2 KO mice were intranasally inoculated with 5 × 10^4^ CFU LVS *clpB*. **(A)** Mice were weighed daily and weight loss is reported as a percentage of starting weight. Mice were sacrificed on days 3, 7, 10, and 14 post-inoculation and bacterial burdens were determined in the **(B)** spleen, **(C)** liver, and **(D)** lung. The dashed line indicates the limit of detection of 50 CFU per organ. Data are combined from two experiments per time point. *n* = 7–10 mice/group. Bacterial burdens were log transformed and then a Student’s *t*-test was used to determine statistical significance.

B6 mice clear LVS *clpB* infection by day 10 post-inoculation (15). We first sought to determine whether disease course and bacterial clearance were altered in TLR2 KO mice inoculated with LVS *clpB*. B6 or TLR2 KO mice were intranasally inoculated with 5 × 10^4^ CFU LVS *clpB* and then sacrificed on days 3, 7, 10, or 14 post-inoculation to determine bacterial burdens. Weight loss profiles in B6 and TLR2 KO mice inoculated with LVS *clpB* differed in peak weight loss (−12% in B6 and −7% in TLR2 KO) and rate of weight gain after day 5 post-inoculation (Figure 1A). Although peak bacteremia was the same in B6 and TLR2 KO mice (day 3 post-inoculation), LVS *clpB* clearance was delayed in the spleen, liver, and lung of TLR2 KO mice (Figures 1B–D). All B6 mice cleared LVS *clpB* by day 14 post-inoculation, whereas only 4 out of 10 TLR2 KO mice had completely cleared LVS *clpB*. The remaining six TLR2 KO mice had low detectable levels of bacteria in the spleen and lung (Figures 1B,D).

### LVS *clpB* vaccination of TLR2 KO mice protects against lethal LVS challenge

**Figure 2:**
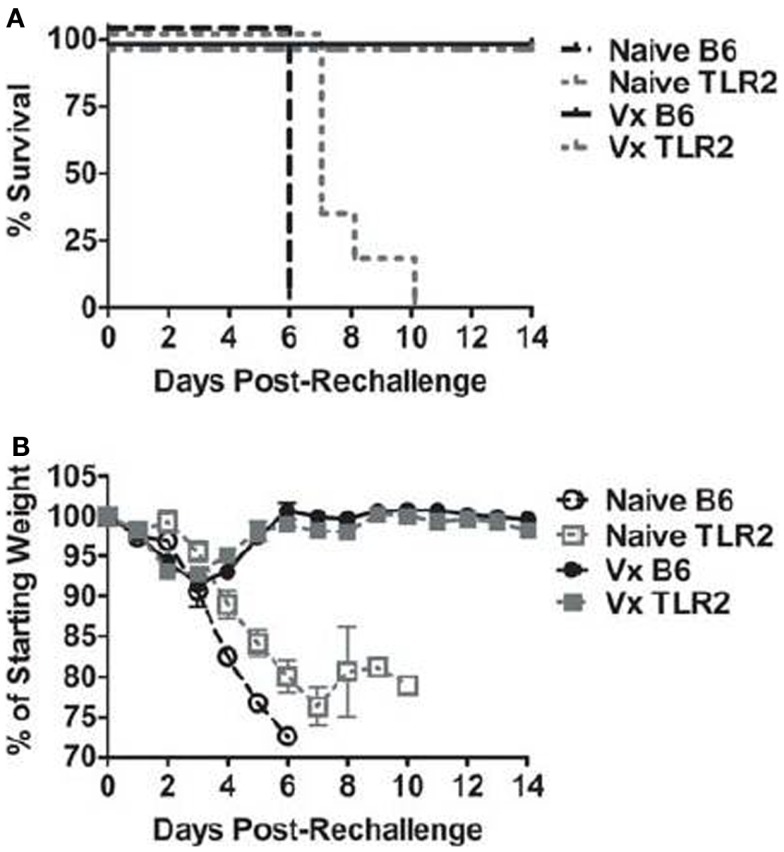
**TLR2 KO previously inoculated with LVS *clpB* survive a lethal LVS challenge**. B6 or TLR2 KO mice were intranasally inoculated with 5 × 10^4^ CFU LVS *clpB* or were left naive. Thirty-five days later, mice were intranasally challenged with 5 × 10^3^ CFU LVS and **(A)** survival was determined. **(B)** Mice were weighed daily and weight loss is reported as a percentage of starting weight. Data are combined from two independent experiments. *n* = 6–10 mice/group.

Prior infection (i.e., vaccination) with LVS *clpB* protects B6 mice during a lethal LVS challenge (15, 20). We therefore sought to determine whether TLR2 was required for protection during a secondary challenge. B6 and TLR2 KO mice were challenged with a lethal dose of LVS 35 days after inoculation with LVS *clpB*. 100% of the B6 and TLR2 KO mice previously inoculated with LVS *clpB* survived the LVS lethal dose challenge, whereas all naïve mice succumbed to infection (Figure 2A). Vaccinated B6 and TLR2 KO mice also had similar weight loss profiles during lethal challenge (Figure 2B). Peak weight loss in both groups occurred on day 3 post-rechallenge and was approximately −8% of the starting weight in both groups (Figure 2B). The weight loss curve for naïve TLR2 KO mice has increased variability because not all mice lost >25% of their starting weight on the same day post-inoculation (Figure 2B). When vaccinated B6 and TLR2 KO mice were sacrificed on day 14 post-rechallenge, no culturable bacteria were present in the spleen, liver, or lung (data not shown), indicating that vaccination with LVS *clpB* provided sterilizing immunity during LVS rechallenge and that TLR2 signaling was not required during this memory response.

### The T-cell response in the lung is similar in B6 and TLR2 KO mice inoculated with LVS *clpB*

**Figure 3:**
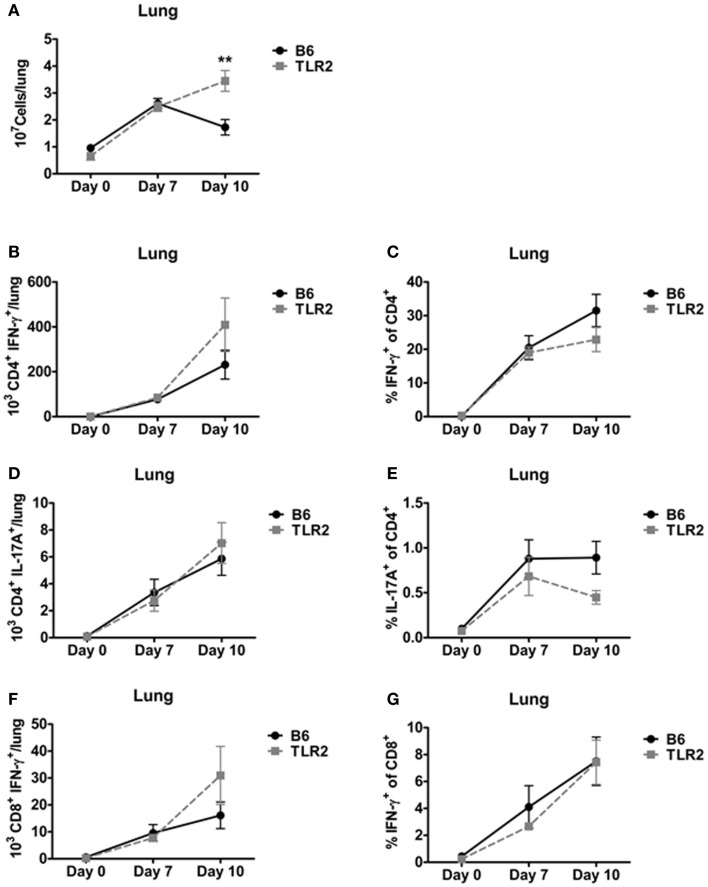
**TLR2 signaling is not required for the T-cell response in the lung**. B6 or TLR2 KO mice were intranasally inoculated with 5 × 10^4^ CFU LVS *clpB* or were left naive. On days 7 and 10 post-inoculation, mice were sacrificed and lungs were removed and digested into a single-cell suspension. **(A)** The total number of cells in the lung was determined by trypan blue exclusion. Lung cells were re-stimulated with LVS-infected CD45.1 splenocytes for 24 h. Brefeldin A was added during the last 4 h of culture. Flow cytometry was used to determine the **(B)** total number of CD4^+^ IFN-γ^+^ T-cells, **(C)** % IFN-γ^+^ of CD4^+^ T-cells, **(D)** total number of CD4^+^ IL-17A^+^ T-cells, **(E)** % IL-17A^+^ of CD4^+^ T-cells, **(F)** total number of CD8^+^ IFN-γ^+^ T-cells, and **(G)** % IFN-γ^+^ of CD8^+^ T-cells. Data are combined from at least two independent experiments per time point. *n* = 4–6 mice/group. Statistical significance was determined using a Mann–Whitney test for each time point.

The ability of TLR2 KO mice to survive a lethal LVS challenge suggested that TLR2-deficient mice are able to mount an effective T-cell response to LVS *clpB*. To determine whether the absence of TLR2 signaling affected the kinetics or magnitude of the T-cell response, we used ICS to enumerate three T-cells subsets (IFN-γ^+^ CD4^+^ (Th1), IL-17A^+^ CD4^+^ (Th17), and IFN-γ^+^ CD8^+^ cytotoxic T-cell) on days 7 and 10 post-inoculation. TLR2 KO mice had a significant increase in lung cellularity compared to B6 mice on day 10, but not day 7, post-inoculation (Figure 3A). The difference observed on day 10 is likely due to the presence of bacteria in TLR2 KO, but not B6 mice. There was no difference in the absolute number of Th1 cells or percentage of IFN-γ^+/^CD4^+^ T-cells in the lungs of LVS *clpB* inoculated B6 or TLR2 KO mice (Figures 3B,C). There was also no difference in the absolute number of Th17 cells in the lung of B6 and TLR2 KO mice or percentage of IL-17A^+^/CD4^+^ T-cells (Figures 3D,E). Finally, there was no difference in the absolute number of IFN-γ^+^ CD8^+^ T-cells or percentage of IFN-γ^+^/CD8^+^ T-cells in B6 or TLR2 KO mice inoculated with LVS *clpB* (Figures 3F,G). Together, these data indicate that the kinetics and magnitude of the T-cell response in the lung during LVS *clpB* infection is similar in B6 and TLR2 KO mice suggesting that TLR2 signaling is not required to mount an adaptive immune response to LVS *clpB*.

### The T-cell response in the spleen is similar in B6 and TLR2 KO mice inoculated with LVS *clpB*

**Figure 4:**
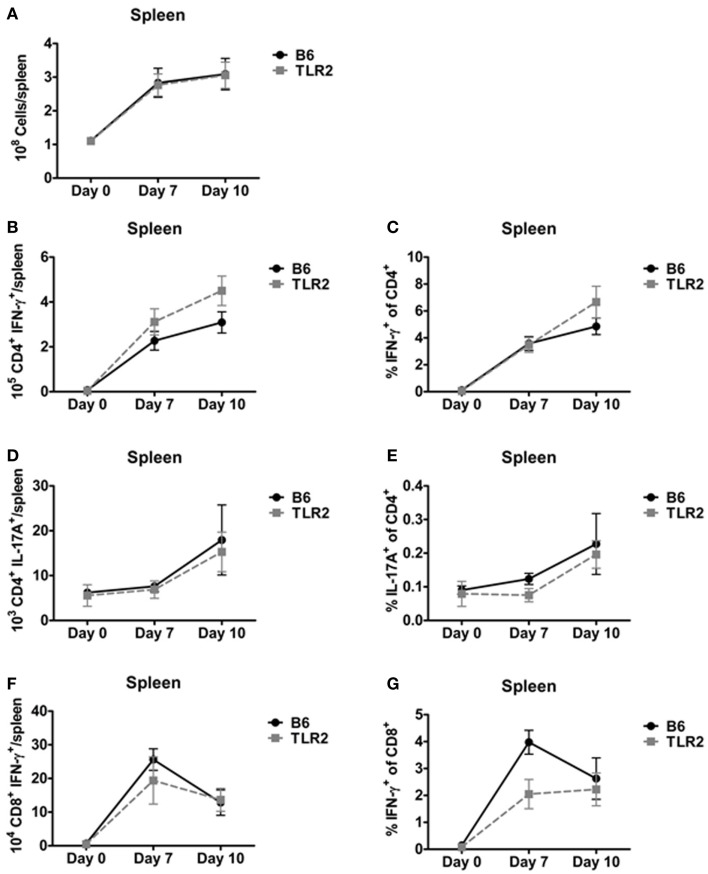
**TLR2 signaling is not required for the T-cell response in the spleen**. B6 or TLR2 KO mice were intranasally inoculated with 5 × 10^4^ CFU LVS *clpB* or were left naive. On days 7 and 10 post-inoculation, mice were sacrificed and spleens were removed and processed into a single-cell suspension. **(A)** The total number of cells in the spleen was determined by trypan blue exclusion. Spleen cells were re-stimulated with LVS-infected CD45.1 splenocytes for 24 h. Brefeldin A was added during the last 4 h of culture. Flow cytometry was used to determine the **(B)** total number of CD4^+^ IFN-γ^+^ T-cells, **(C)** % IFN-γ^+^ of CD4^+^ T-cells, **(D)** total number of CD4^+^ IL-17A^+^ T-cells, **(E)** % IL-17A^+^ of CD4^+^ T-cells, **(F)** total number of CD8^+^ IFN-γ^+^ T-cells, and **(G)** % IFN-γ^+^ of CD8^+^ T-cells. Data are combined from at least two independent experiments per time point. *n* = 4–6 mice/group. Statistical significance was determined using a Mann–Whitney test for each time point.

In addition, we used ICS to identify Th1, Th17, and IFN-γ^+^ CD8^+^ T-cells in the spleen on days 7 and 10 post-inoculation. There was no difference in total spleen cellularity in B6 and TLR2 KO mice inoculated with LVS *clpB* (Figure 4A). There were also no significant differences in the absolute number or percentage of cytokine positive Th1, Th17, or IFN-γ^+^ CD8^+^ T-cell subsets in LVS *clpB* inoculated B6 or TLR2 KO mice (Figures 4B–G). These data indicate that like the lung, the kinetics and magnitude T-cell response in the spleen is similar in B6 and TLR2 KO mice, suggesting that TLR2 signaling is dispensable for the adaptive immune response to LVS *clpB*.

### Cytokine and chemokine production following LVS *clpB* inoculation have differential requirements for TLR2 signaling

**Figure 5:**
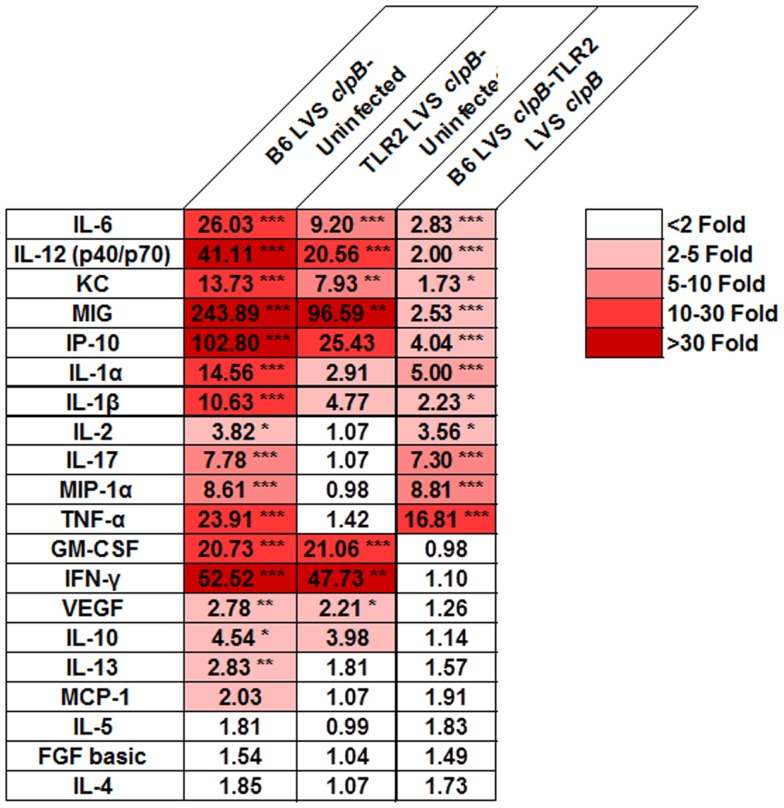
**TLR2 signaling is required for maximal cytokine and chemokine production in the lung after LVS *clpB* inoculation**. B6 or TLR2 KO mice were intranasally inoculated with 5 × 10^4^ CFU LVS *clpB* or were left naive. Three days post-inoculation, mice were sacrificed and BALF was collected, and cytokine and chemokine concentrations were determined using a Luminex-based assay. For each pair of groups, a fold-changed was determined based on the average cytokine or chemokine concentrations. Data are combined from two independent experiments. *n* = 7–12 mice/group. Statistical significance was determined using ANOVA with Tukey’s post-test on the absolute concentration of each analyte.

Although TLR2 appears to be dispensable for the adaptive immune response to LVS *clpB*, we sought to determine whether the innate immune response required TLR2 signaling for maximal cytokine and chemokine production. If so, we can use LVS *clpB* to identify host signaling pathways that are altered during LVS infection, and interrogate the requirement of signaling moieties for the production of specific cytokines and chemokines. Three days post-inoculation with LVS *clpB*, mice were sacrificed and the BALF was collected. The concentration of 20 different cytokines and chemokines in the BALF was determined using a multiplex bead assay and data reported as fold-change (Figure 5). The absolute concentrations of the cytokines and chemokines are listed in Table S1 in Supplementary Material.

We identified three classes of clusters and chemokines: those that were partially dependent on TLR2, those that were dependent on TLR2, and those that were independent of TLR2. Cytokines and chemokines that partially depended on TLR2 signaling for their production are IL-6, IL-12 (p40/p70), KC, MIG, and IP-10. Cytokines and chemokines that depended on TLR2 signaling for their production (i.e., are not made at increased levels in infected TLR2 KO compared to uninfected mice) were IL-1α, IL-1β, IL-2, IL-17, MIP-1α, and TNF-α. GM-CSF, IFN-γ, and VEGF were made at similar levels in B6 and TLR2 KO mice indicating that their production was independent of TLR2 signaling. These data indicated that while TLR2 signaling is responsible for the induction of some cytokines and chemokines, other innate signaling molecules may also contribute to the overall innate response to infection.

### TLR2 is required for maximal cellular infiltration into bronchoalveolar lavage fluid

**Figure 6:**
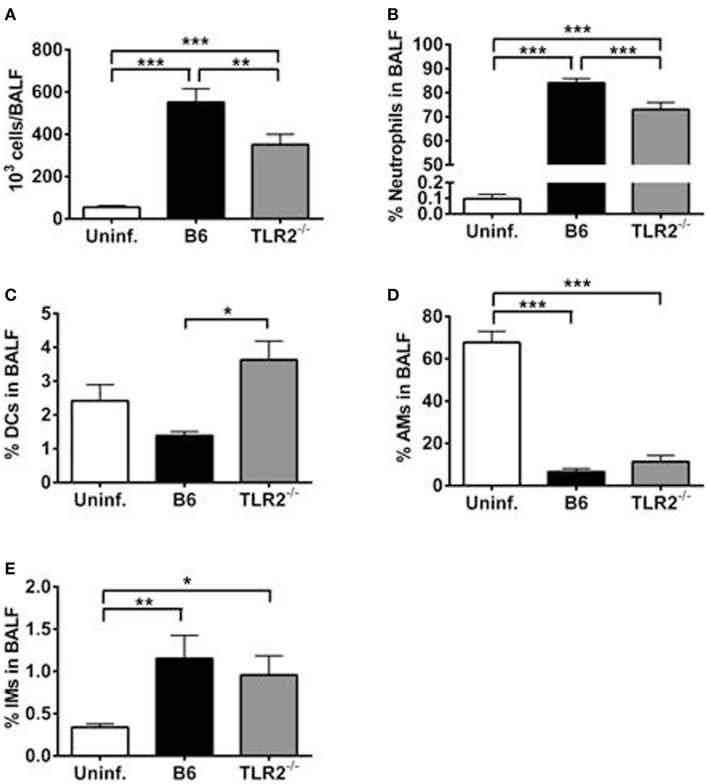
**TLR2 signaling is required for maximal cellular infiltration in the lung after LVS *clpB* inoculation**. B6 or TLR2 KO mice were intranasally inoculated with 5 × 10^4^ CFU LVS *clpB* or were left naive. Three days post-inoculation, mice were sacrificed and BALF was collected and cells removed by centrifugation. **(A)** Total number of cells in the BALF. The % of **(B)** neutrophils, **(C)** dendritic cells (DCs), **(D)** alveolar macrophages (AMs), and **(E)** interstitial macrophages (IMs) in the BALF was determined using flow cytometry. Data are combined from two independent experiments. *n* = 7–12 mice/group. Statistical significance was determined using ANOVA with Tukey’s post-test.

We speculated that the differences observed in BALF cytokine/chemokine milieu between LVS *clpB* inoculated B6 and TLR2 KO mice could impact airspace infiltration by innate immune cells. We therefore used flow cytometry to identify immune cell subsets within the BALF on day 3 post-inoculation. TLR2 KO mice have decreased BALF cellularity compared to B6 mice (Figure 6A). When the cellular composition of the BALF was compared, TLR2 KO mice had fewer neutrophils as a percentage of live cells compared to B6 mice (Figure 6B). TLR2 KO mice had an increased percentage of DCs compared to B6 mice (Figure 6C). There was no difference in the percentage of alveolar macrophages (AMs) or interstitial macrophages (IMs) when LVS *clpB* inoculated B6 and TLR2 KO mice were compared (Figures 6D,E). There was, however, a significant decrease in the frequency of AMs in the BALF of infected animals compared to uninfected control mice (Figure 6D). The frequency of AMs changed in B6 and TLR2 KO mice inoculated with LVS *clpB* because there was an influx of infiltrating neutrophils. When the total number of AMs was compared in uninfected or B6 and TLR2 KO mice inoculated with LVS *clpB*, we did not observe any significant differences between groups (data not shown). Together, these data indicated that differences in the BALF cytokine/chemokine milieu in TLR2 KO mice correlate with changes in BALF cellular composition. Furthermore, these data in conjunction with BALF cytokine and chemokine profiles suggested that TLR2 signaling is required during the innate immune response to LVS *clpB*.

## Discussion

Pattern-recognition receptors, such as TLRs, play a critical role in initiating an innate immune response to microbial pathogens. TLRs except TLR3 and TLR4 require the adaptor protein MyD88 for signaling (5). MyD88-deficient mice are highly susceptible to *Francisella* infection indicating PRRs are critical to the host’s immune response during infection (21). The role of several TLRs has been studied in the context of a *Francisella* infection. Although *Francisella* is a gram-negative pathogen, it has an altered lipid A structure that fails to induce signaling through TLR4 (22–25). TLR4 knock-out (TLR4 KO) mice are not more susceptible than wild-type mice to *Francisella* infection (7, 26, 27). The importance of TLR5 and TLR9 has also been tested in the context of *Francisella* infection, but no phenotype was observed for either PRR (9, 21). To date, TLR2 is the best-characterized PRR for *F. tularensis* (6–13). Pro-inflammatory cytokine production requires TLR2 signaling and TLR2 KO mice are more susceptible to sub-lethal infection with LVS (6–10). In order to evade the TLR2-mediated host immune response, *Francisella* actively inhibits the early innate immune response *in vivo* (14, 15). Lipids derived from SchuS4 inhibit *E. coli* LPS-induced TNF-α and IL-6 production in the lungs of B6 mice, but not TLR2 KO mice indicating that the lipids depend on TLR2 signaling to inhibit the pro-inflammatory response (12). Not only does *Francisella* directly inhibit host signaling via TLR2, it also uses the CRISPR/Cas system to regulate expression of its own bacterial lipoprotein (FTN_1103) that could be sensed by host TLR2 (28).

We have previously shown that LVS *clpB* fails to inhibit the early innate immune response and unlike inoculation with wild-type LVS, a robust pro-inflammatory innate immune response is detected in the BALF on day 3 post-inoculation (15). Despite LVS *clpB*’s attenuation, it elicits a robust T-cell response and previous infection with LVS *clpB* protects 100% of mice challenged with a lethal dose of LVS (15, 20). We were therefore interested in whether TLR2, a key host sensor for detecting *Francisella*, was required during the various phases of the immune response to LVS *clpB*.

Toll-like receptor 2 KO mice exhibited delayed clearance of LVS *clpB* (Figure 1). B6 and TLR2 KO mice had similar peak lung bacterial burdens on day 3 post-inoculation indicating that the delayed clearance was not simply due to an initial increase in bacterial burdens that persists during the course of infection. One possible explanation for the delayed clearance is a delay in the T-cell response in TLR2 KO mice. TLR2 has been shown to be required for CD80, CD86, and MHCII up-regulation in bone marrow-derived DCs inoculated with LVS (8). We did not observe any defects in the T-cell response in TLR2 KO mice on days 7 or 10 post-inoculation, suggesting that a poor T-cell response was not the cause of the delayed bacterial clearance in TLR2 KO mice. Another possible explanation for delayed clearance in TLR2 KO mice is the requirement of both IFN-γ and TNF-α during *Francisella* infection (29–31). Production of IFN-γ during the innate immune response against LVS *clpB* was completely independent of TLR2 signaling (Figure 5). Likewise, TLR2 KO mice intranasally inoculated with wild-type LVS produce significantly more IFN-γ on day 7 post-inoculation compared to B6 mice indicating that TLR2 is not required for IFN-γ production (6). TNF-α production after LVS *clpB* inoculation, however, required TLR2 signaling (Figure 5). TNF-α production in the lungs of TLR2 KO mice inoculated with LVS is delayed and the overall concentration of TNF-α is lower when measured in lung homogenate or by *in situ* TNF-α staining (6, 7). Together, these data indicate that while the IFN-γ-mediated immune response is intact in TLR2 KO mice, there could be defects in the TNF-α-mediated response, which results in delayed LVS *clpB* clearance.

The ability of TLR2 KO mice to survive a lethal LVS secondary challenge suggested that these mice mount a robust adaptive immune response since T-cells are required for survival during a secondary infection (32). Indeed, B6 and TLR2 KO mice had similar absolute numbers and frequencies of Th1, Th17, and CD8^+^ IFN-γ^+^ T-cells in the lung and spleen (Figures 3 and 4) on days 7 and 10 post-inoculation. Our data suggest that TLR2 signaling did not affect the T-cell response during infection with LVS *clpB*. In other infection models, TLR2 KO mice have decreased T-cell responses. For example, when TLR2 KO T-cells are adoptively transferred into wild-type recipients, CD8^+^ T-cells undergo decreased clonal expansion and failed to develop into long-lived memory cells upon vaccina infection (33). In this model, T-cells lacked TLR2 signaling, indicating that TLR2 signaling on T-cells is important during vaccina infection. Although Quigley et al. demonstrated that TLR2 deficiency on T-cell was important, defects in the adaptive immune response observed in the absence of TLR signaling are often attributed to defective antigen presenting cells (34–37). In our model, TLR2 KO mice produce significantly less IL-12, a cytokine required for the polarization of Th1 cells, compared to B6 mice (38). Despite this defect or other defects in TLR2-deficient antigen presenting cells that we did not investigate, the T-cell response in TLR2 KO mice is very similar to the response in B6 mice were TLR2 signaling is intact indicating that TLR2 is dispensable for the adaptive immune response to LVS *clpB*.

We next investigated the requirement of TLR2 during the innate immune response to LVS *clpB*. Because LVS *clpB* fails to inhibit the early innate immune response (15), we could use LVS *clpB* as a tool to identify host signaling pathways that are inhibited during wild-type infection. Intranasal inoculation of B6 and TLR2 KO mice with LVS *clpB* followed by collection of the BALF 3 days post-inoculation, revealed three groups of cytokine and chemokine production: dependent on TLR2, independent of TLR2, and partially dependent on TLR2 (Figure 5). IL-1α, IL-1β, IL-2, IL-17, MIP-1α, and TNF-α production required TLR2 signaling. Notably, the failure of TLR2 KO mice to produce IL-1β suggests that TLR2 signaling provides the first signal that leads to up-regulation of pro-IL-1β mRNA, which is later cleaved by active caspase-1. The requirement of TLR2 signaling for mouse IL-1β production was also demonstrated by Li et al. (9). GM-CSF, IFN-γ, and VEGF production was independent of TLR2 signaling. IL-6, IL-12p40/p70, KC, MIG, and IP-10 were partially dependent on TLR2 signaling. The decreased frequency of neutrophils in the BALF of TLR2 KO mice (Figure 6B) is likely a consequence of less KC (CXCL1) as KC is a chemoattractant for neutrophils (39). TLR2 KO mice produced cytokines that have been shown to be important during LVS infection such as IFN-γ, IL-6, and IL-12 (29–31, 40, 41). The ability of TLR2 KO mice to produce these cytokines, even if at reduced levels compared to B6 mice, indicates that infection with wild-type LVS inhibits other immune signaling pathways in addition to TLR2. The identities of these host sensor(s) are currently unknown.

Overall, we have demonstrated a differential requirement for TLR2 signaling during the innate and adaptive immune response to LVS *clpB*. The T-cell response was similar in B6 and TLR2 KO mice indicating that the adaptive immune response during LVS *clpB* infection does not require TLR2 signaling. TLR2 KO mice also survived a secondary lethal challenge with LVS when first infected with LVS *clpB* indicating that TLR2 is dispensable during the secondary response. Importantly, some cytokines and chemokines were produced in TLR2 KO mice indicating that other signaling pathways are also inhibited during wild-type *Francisella* infection in addition to TLR2. The identities of these pathways are currently unknown, but are a focus of our ongoing research. Together, we have demonstrated that TLR2 is critical during the innate immune response to LVS *clpB* but is not required during the primary or secondary adaptive immune response.

## Author Contributions

Lydia M. Roberts and Hannah E. Ledvina carried out all experiments. Lydia M. Roberts, Gregory D. Sempowski, and Jeffrey A. Frelinger designed experiments and analyzed data. Lydia M. Roberts drafted the manuscript. All authors read and approved the final manuscript.

## Conflict of Interest Statement

The authors declare that the research was conducted in the absence of any commercial or financial relationships that could be construed as a potential conflict of interest.

## Supplementary Material

The Supplementary Material for this article can be found online at http://www.frontiersin.org/Journal/10.3389/fimmu.2014.00426/abstract.
