# Supplementary material for: TLR2 Signaling is Required for the Innate, but Not Adaptive Response to LVS clpB
Source: Front Immunol. 2014 Sep 5;5:426. doi: 10.3389/fimmu.2014.00426 (PMC4155801; doi:10.3389/fimmu.2014.00426)
Supplement: Supplementary file 1 [file Table1.DOCX]

| **Supplemental Table 1.** Mean ± SEM concentration (pg/mL) of cytokines and chemokines from the broncheoalveolar lavage fluid on day 3 post-inoculation | | | |
| --- | --- | --- | --- |
|  | **Uninfected (n=12)** | **B6 *clpB* (n=7)** | **TLR2 *clpB* (n=8)** |
| IL-6 | 15.46 ± 0.36 | 402.50 ± 37.18 | 142.27 ± 15.70 |
| IL-12 (p40/p70) | 3.46 ± 0.09 | 142.14 ± 13.59 | 71.08 ± 9.14 |
| KC | 76.12 ± 17.09 | 1003.56 ± 145.04 | 579.44 ± 128.80 |
| MIG | 11.06 ± 2.51 | 2697.88 ± 355.73 | 1068 ± 142.52 |
| IP-10 | 12.56 ± 0.93 | 1291.03 ± 178.22 | 319.30 ± 50.12 |
| IL-1α | 12.75 ± 0.13 | 185.69 ± 19.46 | 37.13 ± 11.40 |
| IL-1β | 12.31 ± 0.31 | 130.79 ± 20.93 | 58.69 ± 22.06 |
| IL-2 | 5.47 ± 0.71 | 20.90 ± 7.30 | 5.88 ± 0.80 |
| IL-17 | 5.29 ± 0.61 | 41.15 ± 9.95 | 5.64 ± 0.69 |
| MIP-1α | 17.39 ± 0.71 | 149.77 ± 17.25 | 17.00 ± 0.80 |
| TNF-α | 11.55 ± 0.03 | 276.02 ± 26.05 | 16.42 ± 4.33 |
| GM-CSF | 9.98 ± 0.22 | 206.93 ± 16.61 | 210.23 ± 21.47 |
| IFN-γ | 10.86 ± 2.07 | 570.32 ± 117.24 | 518.31 ± 123.68 |
| VEGF | 37.36 ± 4.24 | 103.97 ± 18.22 | 82.72 ± 13.03 |
| IL-10 | 90.52 ± 27.24 | 411.29 ± 116.91 | 360.02 ± 109.89 |
| IL-13 | 15.21 ± 1.46 | 43.13 ± 6.97 | 27.54 ± 6.07 |
| MCP-1 | 16.15 ± 1.89 | 32.81 ± 10.49 | 17.21 ± 2.12 |
| IL-5 | 9.83 ± 0.19 | 17.76 ± 6.96 | 9.73 ± 0.21 |
| FGF basic | 20.99 ± 1.36 | 32.38 ± 10.62 | 21.75 ± 1.53 |
| IL-4 | 33.07 ± 3.88 | 61.01 ± 26.39 | 35.25 ± 4.35 |
